# Amoeboma of the gallbladder mimicking a cholangiocarcinoma: A case report

**DOI:** 10.1016/j.ijscr.2023.108656

**Published:** 2023-08-11

**Authors:** Yacine Ouadi, Mahdi Hammami, Wassim Frikha, Heifa Kamoun, Fadhel Fterich, Montasser Jameleddine Kacem

**Affiliations:** a Department of Surgery A La Rabta Hospital, Tunis, Tunisia; b Faculty of medicine of Tunis, Tunis El Manar University, Tunis, Tunisia; c Department of radiology La Rabta hospital, Tunis, Tunisia; d Department of pathology La Rabta hospital, Tunis, Tunisia

**Keywords:** Case report, Amoeboma, Cholangiocarcinoma, General surgery

## Abstract

**Introduction and importance:**

Amoeboma is a pseudotumoral presentation of amebiasis which is a parasitic infection caused by entamoeba histolytica. Its location in the gallbladder is extremely rare. Indeed, only one other case was found in the literature. Therefore, we present this case report on managing a gallbladder amoeboma mimicking a cholangiocarcinoma.

**Case presentation:**

A 62-year-old presenting for consultation for biliary colic that has been developing for 4 months without associated signs.

MRI and thoraco-abdominal CT concluded to a cholangiocarcinoma of the gallbladder extended to the liver with probable localized peritoneal carcinosis.

We, therefore, performed extended cholecystectomy with lymphadenectomy for the diagnosis of cholangiocarcinoma.

Pathology concluded to an amoeboma of the gallbladder extended to the liver and duodenum.

**Clinical discussion:**

To our knowledge, there is only one case of gallbladder amoeboma in the literature making this case report valuable.

It is important to draw lessons of this observation. Indeed, in front of the discrepancy between the clinic, biology (good general condition and negative tumor markers) and the imaging, we prefer this therapeutic strategy: make a biopsy of the hepatic parenchyma, realize amoebic serology to confirm the diagnosis. Then subject the patient to a therapeutic test based on metronidazole and confirm the disappearance of suspicious lesions by CT scan.

**Conclusion:**

Gallbladder amoeboma is an exceptional entity, but it needs to be kept in mind in case of an atypical presentation of a cholangiocarcinoma. Evoking and confirming the diagnosis preoperatively makes it possible to avoid excessive surgery.

## Highlights

• Amoeboma is a pseudotumoral presentation of amebiasis.
• Amoeboma of the gallbladder is extremely rare.
• Ameoboma of the gallbladder can mimic a cholangiocarcinoma.
• Surgery is not necessary if a preoperative diagnosis is made.

## 1 Background

Amoeboma is a pseudotumoral presentation of amebiasis which is a parasitic infection caused by entamoeba histolytica. It can occur in the colon mimicking colonic cancer or in the liver mimicking a liver tumor (1). its location in the gallbladder is extremely rare. Therefore, we present this case report on managing a gallbladder amoeboma mimicking a cholangiocarcinoma.

This case report has been reported in line with the SCARE Criteria (2).

## 2 Case presentation

A 62-year-old patient with no medical history, with a surgical history of surgery on both knees 20 years ago, presenting for consultation for biliary colic that has been developing for 4 months without associated signs.

**Fig. 1:**
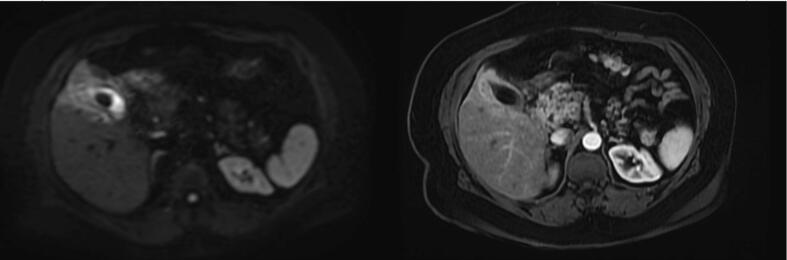
Irregular fundal gallbladder wall thickening with invasion of adjacent liver. It presents a high signal in T2 fat sat sequences with moderate contrast enhancement. Note the gallbladder lithiasis in low T2 signal.

The examination found a patient in good general condition, without palpable mass. Biology was without any abnormalities. Abdominal ultrasound showed a suspicious thickening of the gallbladder with vesicular lithiasis. The MRI showed a lithiastic gallbladder with irregular 14.5 mm parietal thickening budding in the gallbladder fundus, extended over 5 cm and infiltrating segments IV and V of the liver with moderate infiltration of surrounding tissues. There is also one lymphadenopathy of the liver hilum of 12mmx17 mm (Fig. 1).

All evoking cholangiocarcinoma of the gallbladder extended to the liver with probable localized peritoneal carcinosis.

Considering the suspicion of gallbladder tumor, we completed with tumor markers that came back normal (ACE <1.73 μg/L and Ca19-9 34 U/ml) and with a thoraco-abdominal CT that confirmed the findings of the MRI and found no other secondary locations.

We, therefore, decided to perform extended cholecystectomy with lymphadenectomy for the diagnosis of cholangiocarcinoma.

The patient underwent surgery on 11-05-2022 through a right subcostal incision, we found inflammatory-looking adhesions between the epiploon and the gallbladder and between the duodenum and the gallbladder. There were also multiple centimetric lymphadenopathies behind the portal vein and along the hepatic artery.

**Fig. 2:**
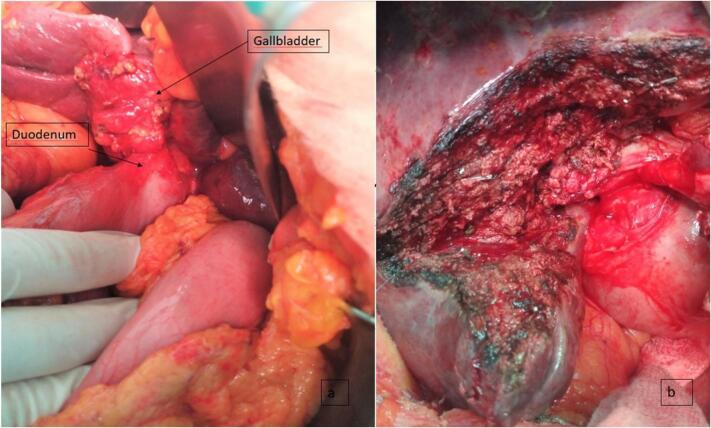
a: Picture showing the gallbladder retracting the hepatic parenchyma and the duodenum. b: Picture showing the operative site after the IVb-V bisegmentectomy with lymphadenectomy.

The gallbladder was indurated in its entirety retracting the hepatic parenchyma and the duodenum. There were no liver metastases or peritoneal carcinosis (as suspected preoperatively) (Fig. 2a).

The omentum is therefore sectioned, and a duodenal collar is removed by means of a TA blow so as not to open the gallbladder. A IVb-V bisegmentectomy is performed with extended lymphadenectomy (Fig. 2b). we decide to keep the tran-cystic drain since there was a bile leak that we controlled by sutures after the end of the hepatectomy.

The surgical site is drained.

Post-operative follow-up was complicated by an infected subhepatic billoma which evolved well with percutaneous drainage and antibiotics and the patient was discharged on day 20 postoperatively with drains in place that were removed one month later.

**Fig. 3:**
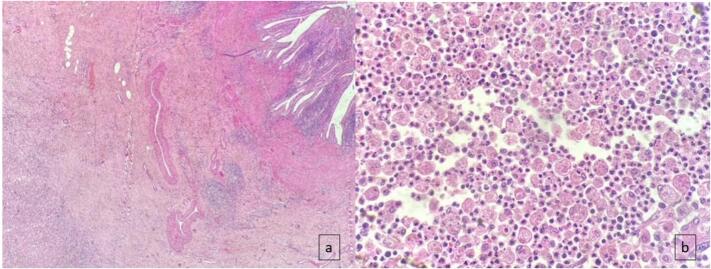
a: Low magnification (×100) of the duodenal mucosa with the presence of an abscessed focus in the wall containing amoebae, polymorphonuclear neutrophils, lymphocytes. It is associated with hemorrhagic diffusions. b: Appearance of amoebas at high magnification (×400) where the flaky structures are with a pale cytoplasm containing red blood cells and a repressed nucleus.

Pathology concluded to an amoeboma of the gallbladder extended to the liver and duodenum (Fig. 3).

Patient received one tablet three times a day of metronidazol 500 mg for two months.

A postoperative CT scan was conducted two months after surgery and was normal.

## 3 Discussion

Amoeboma is caused by *Entamoeba histolytica*. This parasite becomes invasive in only 10 % of cases. Thus, compared to the number of people carrying amoebas, the disease is relatively rare and does not occur in most cases. In invasive amoebiasis, after ingestion of cysts (food, water, dirty hands, fecal peril), the amoeba enters the submucosa and causes enteritis. If the invasive phenomenon continues, the parasites can be found by breaking into the mesenteric venules and, stopped at the level of the liver, determine hepatic tissue amoebiasis or amoebic abscess (5 to 10 % acute intestinal amoebiasis, more often in men and more often in the right lobe) potentially severe in the absence of prompt treatment. Much more rarely, amoebae can pass the hepatic filter and locate in any other organ (3).

Amoeboma is a pseudotumoral presentation of amebiasis. It can occur in the colon mimicking colonic cancer or in the liver mimicking a liver tumor. Colonic amoeboma is the most frequent localization (1). The liver is the most common extra enteral localization for amoebiasis (4).

To our knowledge, there is only one case of gallbladder amoeboma in the literature (4) making this case report valuable. This exceptional location does not offer specific symptomatology, indeed both our patient and the one in the literature presented biliary type pain. Pre-operative lab work did not find abnormalities. Imaging devices are likely to find an aspect of cholangiocarcinoma of the gallbladder.

The sanction of these findings is a surgical resection that is usually a IVb-V bisegmentectomy with lymphadenectomy to perform a curative procedure which was done in both our case and the one in the literature.

Furthermore, in our case, the lesion was locally advanced with preoperative signs mimicking a localized peritoneal carcinosis. This made the decision for an exploratory laparotomy questionable.

What we also found during the operation was very interesting as the gallbladder lesion invaded the surrounding tissues with intimate contact with the omentum and duodenum. These adhesions simulate rather an inflammatory origin than a tumoral invasion.

Although intraoperative aspects are more reminiscent of inflammatory lesions, we preferred to treat as cholangiocarcinoma to not miss the opportunity for curative surgery. This attitude is more legitimate since the patient has a large gallbladder stone which is a known risk factor for this pathology.

It is important to draw lessons of this observation. Indeed, in front of the discrepancy between the clinic, biology (good general condition and negative tumor markers) and the imaging, we prefer this therapeutic strategy: make a biopsy of the hepatic parenchyma, realize amoebic serology to confirm the diagnosis. Then subject the patient to a therapeutic test based on metronidazole and confirm the disappearance of suspicious lesions by CT scan.

## 4 Conclusion

Gallbladder amoeboma is an exceptional entity, but it needs to be kept in mind in case of an atypical presentation of a cholangiocarcinoma. Evoking and confirming the diagnosis preoperatively makes it possible to avoid excessive surgery.

## Provenance and per review

Not commissioned, externally pee-reviewed.

## Consent

Written informed consent was obtained from the patient for publication of this case report and any accompanying images. A copy of the written consent is available for review by the Editor-in-Chief of this journal on request.

## Availability of data and materials

All data are available for review.

## Ethical approval

The article is a case report that does not require ethical approval in our country to be published, only patient consent is necessary. The study is exempt from ethnical approval in our institution.

## Funding

No sources of funding.

## Author contribution

All authors contributed to the work.

## Guarantor

The corresponding author.

## Research registration

Not applicable.

## Declaration of competing interest

All authors declare they have no conflict of interest.
